# *Scrophularia Tenuipes* Coss and Durieu: Phytochemical Composition and Biological Activities

**DOI:** 10.3390/molecules25071647

**Published:** 2020-04-03

**Authors:** Zeyneb Chaibeddra, Salah Akkal, Houria Ouled-Haddar, Artur M. S. Silva, Ammar Zellagui, Mohamed Sebti, Susana M. Cardoso

**Affiliations:** 1 Laboratory of Molecular Toxicology, Faculty of Nature and Life Science, University of Mohamed Seddik Benyahia, 18000 Jijel, Algeria; porphyrie2010@yahoo.fr (Z.C.); houledhaddar@univ-jijel.dz (H.O.-H.); 2 Laboratory of Phytochemistry and Physical-Chemical and Biological Analyses, Department of Chemistry, Faculty of Exact Sciences, University of Mentouri Constantine, 25000 Constantine, Algeria; salah4dz@yahoo.fr; 3 LAQV-REQUIMTE Department of Chemistry & Organic Chemistry, Natural Products and Food Stuffs Research Unit, University of Aveiro, 3810-193 Aveiro, Portugal; artur.silva@ua.pt; 4 Laboratory of Biomolecule and Plant Breeding, Life Science and Nature Department, Faculty of Exact Science and Life Science and Nature, University of Larbi Ben Mhidi, 4000 Oum El Bouaghi, Algeria; zellaguiuniv@yahoo.com; 5 Laboratory of Biotechnology Environment and Health, University of Mohamed Seddik Benyahia, 18000 Jijel, Algeria; lbes@univ-jijel.dz

**Keywords:** *Scrophularia tenuipes*, UHPLC-ESI-DAD-MS^n^, phenolic, iridoids, phenylethanoid, anti-inflammatory, antioxidant, antidiabetic

## Abstract

*Scrophularia tenuipes* is an Algerian-Tunisian endemic species, which has not been studied yet. Ethyl acetate (EA) and *n*-butanol (Bu) fractions obtained from *Scrophularia tenuipes* were investigated for their health benefit properties, in particular with respect to in vivo/in vitro anti-inflammatory and antioxidant activities, as well as their potential to inhibit key enzymes with impact in diabetes (α-glucosidase and α-amylase). The fractions had a distinct phytochemical composition, of which EA was richer in total phenolic compounds (225 mg GAE/g) and mostly composed of the phenylethanoid acetyl martynoside. Compared to EA, Bu had higher amounts of total flavonoids, and according to the result obtained from UHPLC-DAD-ESI-MS^n^ analysis, harpagoside (iridoid) was its major phytochemical. EA fraction was quite promising with regard to the in vivo (at 200 mg/kg, po) anti-inflammatory effect (62% and 52% for carrageenan-induced rat paw edema and xylene-induced ear edema tests, respectively), while Bu fraction exhibited a stronger antioxidant capacity in all tests (IC_50_ = 68 µg/mL, IC_50_ = 18 µg/mL, IC_50_ = 18 µg/mL and A_0.50_ = 43 µg/mL for DPPH^●^, ABTS^•+^, O_2_^•−^ scavenging assays and cupric-reducing antioxidant capacity method, respectively). Both fractions also showed a strong effect against α-amylase enzyme (IC_50_ = 8 µg/mL and 10 µg/mL for EA and Bu fraction, respectively).

## 1. Introduction

*Scrophularia* genus (Scrophulariaceae) includes about 350 species commonly known as figwort, from which 11 are represented in Algeria. In general, these plants grow wild in nature, mainly occurring in mountainous regions [1,2]. Many *Scrophularia* plants are used in traditional medicine for the treatment of fever, erythema, eczema, wounds, inflammation of skin, dermatosis, ulcers, abscesses, fistulas, and cancer [3], with most of the health claims associated with their richness in second metabolites. In fact, previous studies focusing on distinct species of this genus remarked on the presence of phenylpropanoids, phenylethanoids, phenolic acids, flavonoids, iridoid glycosides, and terpenoids [4,5,6,7,8,9,10], which are known to exhibit distinct biological properties [4,11]. Such evidence has been highlighted by Ahmed et al., who demonstrated that the iridoid glycosides scropolioside-D2 and harpagoside, both isolated from *S. deserti,* exhibited anti-inflammatory and antidiabetic effects in mice models [12]. Likewise, among several compounds isolated from *S. koelzii*, the iridoid scropolioside-A was shown to possess powerful hepatoprotective activity against thioacetamide-induced hepatotoxicity in an animal model [13]. Moreover, in the work of Ahmed et al. [12], the phenylethanoid glycosides acetoside and martynoside, isolated from *S. deserti*, were described as good antioxidant compounds, while the phenylpropanoid scropheanoside-III was demonstrated to exert relevant in vivo anti-inflammatory activity. Besides, Kim et al. [14] demonstrated that acacetin isolated from *S. takesimensis* had strong aldose reductase inhibitory activity. Additionally, phenolic acids, such as *m*- and *p-*methoxycinnamic acid and ferulic acid, isolated from *S. canina*, were shown to ameliorate carbon tetrachloride-induced hepatotoxicity in animal tests [15,16].

*Scrophularia tenuipes* Coss and Durieu is an endemic threatened species. The plants are perennial and characterized by very loose inflorescences and small and numerous flowers. They occur at four sites in Tunisia (Ain Draham, Kroumirie Babouch, valley Mellegue, and valley of the Medjerda Ghardimaou) and in Algeria, where they have widespread to Great Kabylie, mainly Bouira and Tizi-Ouzou (in the mountains of Djurdjura), and Little Kabylie, mostly Bejaia, Jijel, and Setif (in the Babors mountains), besides other cities, such as Skikda, Annaba, and El-Taref. The plants of this species grow in wet or marshy places at the edge of springs and streams [17,18]. Based on an ethnobotanical investigation conducted in Algeria, *S. tenuipes* is commonly used as an infusion form in folk medicine to treat inflammation, hemorrhage, and diabetes, among other health problems [19,20,21]. However, as far as we know, the phytochemical composition and beneficial effects of this species remain unexplored. Hence, the present study aimed to evaluate the biological effects of the aerial parts of *S. tenuipes*, mainly focusing on their potential anti-inflammatory and antioxidant activities, and inhibitory capacities towards key metabolic enzymes with impact in diabetes, while relating those with their major bioactive components.

## 2. Results and Discussion

### 2.1. Phenolic Compounds of Ethyl Acetate (EA) and n-Butanol (Bu) Fractions

**Figure 1:**
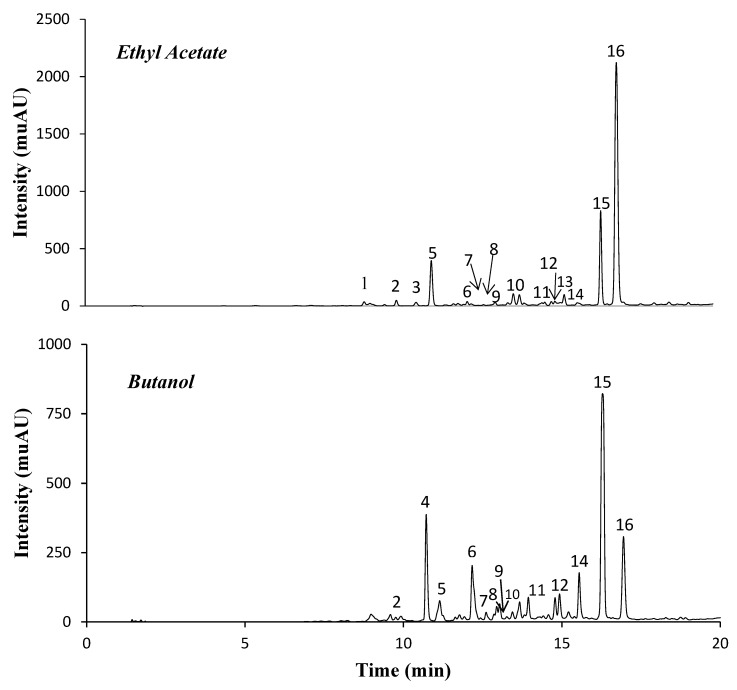
Chromatographic representation of *Scrophularia tenuipes* fractions at 280 nm. Numbers in the figure correspond to the UHPLC-DAD-ESI-MS^n^ peaks described in Table 1.

The EA and Bu fractions obtained from *S. tenuipes* represented 0.15% and 0.32% of the dried plant, respectively. Notably, the two fractions differed significantly regarding their contents in total phenolic compounds (TPCs) and total flavonoids (TFs), with EA presenting superior levels of TPCs but less flavonoids than Bu (Table 1). Their distinct composition was also confirmed by UHPLC-DAD-ESI-MS^n^ analysis (Figure 1 and Table 1). In fact, the main chromatographic peak (peak 16) in EA corresponded to a pseudomolecular ion at *m*/*z* 693, while the major peaks in Bu (peaks 14 and 15) showed [M − H]^−^ at *m*/*z* 493. The [M − H]^−^ at *m*/*z* 693 was assigned to the phenylethanoid acetyl martynoside based on its fragmentation pattern, which gave an ion at *m*/*z* 651 (corresponding to martynoside) as a consequence of the loss of 42 Da (i.e., an acetyl moiety) and at *m*/*z* 633, the last being the result of an additional loss of water (−18 Da). Moreover, the MS^2^ spectrum of this compound showed several product ions also arising from the break of the martynoside moiety. In particular, the ion at *m*/*z* 505 [M – H – 42 − 146]^−^ resulted from the simultaneous loss of the acetyl and of a rhamnosyl moiety; the ions at *m*/*z* 517 [M – H − 176]^−^ and 475 [M – H – 42 − 176]^−^ corresponded to the loss of the feruloyl moiety or of its combined loss with an acetyl group, and the ion at *m*/*z* 457 could arise from the latter by an additional loss of water [22]. In turn, the [M − H]^−^ at *m*/*z* 493 in peaks 14 and 15 were attributed to two harpagoside isomers, also based on their fragmentation pattern and literature data [23]. Their MS^2^ spectra showed an ion at *m/z* 345 (−148 Da), which resulted from the loss of the cinnamoyl moiety from the deprotonated ion. In addition, the characteristic ions at *m/z* 147 confirmed the existence of a cinnamoyl moiety and the [M – H – cinnamoyl − 162] ion (at *m/z* 183) was consistent with the elimination of a glucose moiety.

In accordance with the results obtained from the total flavonoids assay, kaempferol derivatives were predominant in the Bu fraction. These corresponded to two kaempferol-*O*-hexoside isomers (peaks 6 and 8), as confirmed by the presence of the aglycone kaempferol (ion at *m*/*z* 285 in MS^2^ spectra) and the [M − H]^−^ at *m*/*z* 447→285 (loss of 162 Da, i.e., loss of a hexoside moiety) and to kaempferol-*O*-dihexoside (peak 4), [M−H]^−^ at *m*/*z* 609→447, 285 (−162 Da and −324, i.e., loss of one or two hexoside moieties, respectively). In addition, the EA and Bu fractions presented several coumaric acid derivatives (characteristically showing ions at *m*/*z* 163 and 119 in MS^2^ spectra), which appeared as minor components. Globally, these were eluted in peaks 2, 7, and 9–13, and, with exception of [M − H]^−^ at *m*/*z* 509 (peak 7), were assumed to have acetyl and/or rhamnose moieties in their structure. In particular, the fragmentation pattern of [M − H]^−^ at *m*/*z* 309 in peak 2 was consistent with coumaroyl-*O*-rhamnoside since the ion at *m*/*z* 163 (i.e., coumaric acid) resulted from the loss of 146 Da (i.e., rhamnosyl moiety). Instead, the formation of *m*/*z* 163 in [M − H]^-^ at *m*/*z* 351 (peaks 9, 10, and 13) was due to the loss of 188 Da, which could correspond to the combined loss of an acetyl and a rhamnosyl unit. In turn, compounds of [M − H]^−^ at *m*/*z* 679 (peaks 11 and 12) were considered as derivatives of coumaroyl-*O*-acetyl-rhamnoside. In this case, the product ions at *m*/*z* 637 and at *m*/*z* 499 corresponded to the loss of acetyl and rhamnosyl units, respectively, while the product ion at *m*/*z* 351 (i.e., coumaroyl-*O*-acetyl-rhamnoside) resulted from the loss of 180 Da.

In general, the phytochemical compounds herein found in the two *S. tenuipes* fractions are in line with previous reported data for *Scrophularia* plants, since phenylethanoid/phenylpropanoid glycosides and iridoids represent their major metabolites. In this regard, martynoside has been previously isolated from *S. dentata* [1], *S. xanthoglossa* [24], *S. koraiensis* [25], and *S. umbrosa* [26], while the iridoid harpogoside was reported for *S. ningpoensis* [6], *S. scorodonia* [7,27], *S. buergeriana* [8], and *S. nodosa* [5]. Additionally, caffeic acid, coumaric acid derivatives, and kaempferol derivatives were also previously detected in *Scrophularia* plants. In more detail, coumaric acid and/or caffeic acid were reported in *S. frutescens*, *S. sambucifolia*, *S. buergeriana, S. nodosa* L. [5,28,29], *S. kotscyhana*, *S. cinarescens*, *S. catariifolia*, *S. chrysantha*, and *S. scopolii* [10]; and kaempferol and/or some of its glycosidic derivatives were described, among others, in *S. ilwensis*, *S. lucida*, *S. nodosa* L., and *S. scopolii* [5,30,31].

### 2.2. Anti-Inflammatory Activity

Inflammation is a complex physiological response of living tissues to injury and is known to involve a complex array of enzymes’ activation, mediators’ release, extravasations of fluid, cell migration, tissue breakdown, and repair [32]. In this respect, protein’s denaturation is also pointed as a central cause of inflammation [33]. The potential anti-inflammatory activity of *S. tenuipes* EA and Bu fractions was monitored in two widely used rat models, namely carrageenan-induced paw edema and xylene-induced ear edema models. Moreover, the ability to counteract protein denaturation was estimated as part of the investigation on the mechanism of the anti-inflammation activity.

As summarized in Table 2, the injection of the rats with carrageenan (1%) caused a paw edema, which was characterized by a volume of 2.4 mL upon 1 h and tended to decrease over time, reaching 1.4 mL at 3 h, in control conditions. The pre-supplementation of the animals’ diet with *S. tenuipes* fractions, at a dose of 200 mg/kg, considerably prevented inflammation. The major preventive effect was obtained with EA fraction, for which the paw-edema volume was reduced by approximately half in the first 2 h and by 63% at the third hour, as compared to the control. Although less effective, the Bu fraction also prevented the paw edema of the rats over the experiment time (18%, 21%, and 23% as compared with the control at 1, 2, and 3 h, respectively). Please note that edema formation is a result of complex interactions among various inflammatory mediators that contribute to the increase of vascular permeability and/or blood flow. In the case of the carrageenan-induced edema, this occurs as a biphasic event, in which the early phase, observed about 1 h after carrageenan injection, is due to the release of the modulators serotonin and histamine, while the late phase occurs due to the release of bradykinin, protease, prostaglandin, and lysosome, meaning that suppression of the early phase and late phases may be due to inhibition of the release of early mediators and by inhibition of cyclo-oxygenase, respectively [34]. Based on this, our results suggest that the inflammatory potential of *S. tenuipes* fractions occurs by both these mechanisms.

Xylene-induced ear edema is frequently used as an acute inflammation model. Ear-edema formation may involve a release of inflammatory mediators, promoting vasodilation and increasing vascular permeability, and these mediators can induce ear edema [35]. The anti-inflammatory capacity of the *S. tenuipes* EA and Bu fractions and the superior potential of the first were also evidenced in the xylene-induced ear edema model. In fact, rats administered with EA and Bu at 200 mg/kg showed an ear-edema suppression of 53% and 25% compared to the control group, respectively. This result further suggests that *S. tenuipes* fractions may produce anti-inflammatory effects through inhibition of the inflammatory mediators of the acute phase of inflammation. As regards the ability to impair protein denaturation processes, our results showed that this event was also rather inhibited by *S. tenuipes* EA (this fraction inhibited 81% at 2 mg/mL while Bu was ineffective).

Hence, the overall gathered results support the anti-inflammatory claims of *Scrophularia* plants and of *S. tenuipes*. In addition, considering the richness of EA in total phenolic compounds and in particular of acetyl martynoside, it is possible to suggest that this is a critical metabolite for the anti-inflammatory potential of *S. tenuipes*. Martynoside isolated from the Scrophulariaceae family was reported to have a remarkably anti-inflammatory activity: when isolated from the genus *Verbascum salviifolium* and tested on a carrageenan-induced hind paw edema in mice, it exerted a significant anti-inflammatory potential (60% inhibition) [36]. In the same animal model, martynoside combined with aquaticoside A and aquaticoside B from *Veronica anagallis-aquatica* showed an inhibitory capacity of 48.9% [37].

However, it is worth mentioning that other components, particularly harpagoside, are expected to contribute to the anti-inflammatory ability of the two fractions. Indeed, harpagoside is widely recognized as an anti-inflammatory agent and its content is identified as an anti-inflammatory marker in the *Harapgophytum procumbens* (Devil’s claw) root extracts, which are a common ingredient in nutraceutical products for the treatment of inflammation due to arthritis [38]. The fact that the EA fraction showed a superior anti-inflammatory ability over Bu suggests that acetyl martynoside may exert a superior ability to counteract key inflammatory mechanisms occurring in the carrageenan-induced edema and xylene-induced ear edema models, as well as to act against protein denaturation. Another possibility may be due to the presence of other compounds in the Bu fraction that can neutralize the anti-inflammatory activity of harpagoside. Naturally, further studies with isolated compounds from EA and Bu must be performed to better clarify the biological potency of its components.

To our knowledge, distinct species of the *Scrophularia* genus have an anti-inflammatory potential. In this context, the iridoids (scropolioside B and scropolioside D) isolated from *S. denata* were shown to exhibit anti-inflammatory effects in an NF-κB-mediated reporter gene luciferase assay [39]. Verbascosaponin isolated from *S. auriculata* strongly inhibited the carrageenan paw edema and ear edema induced by 12-*O*-tetradecanoylphorbol 13-acetate (TPA test) [40]. According to Fernandez et al., several phenolic acids isolated from *S. frutescens*, including ferulic, gentisic, protocatechuic, and syringic acids, were active in the TPA test [29]. Moreover, the ethyl acetate fraction of *S. striata* was shown to inhibit IL-1b, TNF-a, and prostaglandin E2 (PGE2) secretion in mouse peritoneal macrophages induced by lipopolysaccharide (LPS) [41].

### 2.3. Antioxidant Activity

Oxidative stress has been implicated in numerous pathologic conditions, such as inflammation, diabetes, cardiovascular diseases, cancer, and ageing [42], while natural fractions are potential sources of bioactive compounds able to counteract such events. In this work, *S. tenuipes* EA and Bu fractions were screened for their antioxidant abilities through their ability to scavenge DPPH, ABTS, and superoxide radicals, as well as the ability to reduce cupric ion (Table 3). Both fractions exhibited considerable antioxidant potential and, curiously, regardless of the lower content of phenolic compounds, Bu was in general more effective than EA, a fact that was particularly evident in the DPPH^●^ (IC_50_ 68.9 and 111.1 µg/mL, respectively) and CUPRAC (A_0.50_ of 43.2 and 53.3 µg/mL, respectively) assays. Considering the distinct phenolic composition of the fractions, one may hypothesize that the superior antioxidant potential of Bu can in part be associated to its richness in flavonoids and/or the iridoid harpagoside, both previously pointed out to be effective antioxidants. In fact, harpagoside isolated from *S. buergeriana* was shown to possess high antioxidant power in a scopolamine-treated mice model [43].

### 2.4. α-Glucosidase and α-Amylase Assay

An unexpected increase of glucose levels in blood causes hyperglycemia in type-2 diabetes patients due to hydrolysis of starch by pancreatic α-amylase and the consequent absorption of glucose by intestinal α-glucosidase [44]. Thus, the strong inhibition of α-glucosidase and mild inhibition of pancreatic a-amylase is believed to be an effective strategy for type-2 diabetes management [45]. Although the two fractions showed no promising α-glucosidase inhibitory effects (Table 4), their potency to suppress α-amylase activity was stronger than that of the commercial drug acarbose (IC_50_ value 8.3, 10.98, and 45.20 µg/mL for EA, Bu, and acarbose, respectively). Among the two fractions, EA was the most promising one (about 5.5-fold the potency of acarbose), a fact that may also be associated to its phenolics richness and in particular to its major phenolic compound, i.e., acetyl martynoside, or even to phenolic synergies, as suggested by McCue and Shetty [46]. As far as we know, the in vivo antidiabetic ability of *Scrophularia* plants and the simultaneous association to α-amylase inhibitory activity was previously reported by Ahmed et al. for *S. deserti* [12]. Consistent with our results, *S. frigida* methanolic and aqueous fractions showed weak inhibitory effects towards α-glucosidase [47].

## 3. Materials and Methods

### 3.1. Chemicals

Kaempferol, coumaric acid, and verbascoside, minimum 95% purity, were obtained from Extrasynthese (Genay Cedex, France). Other solvents and reagents were of analytical grade and were either Sigma (St Louis, MO, USA) or Merck Life Science (Darmstadt, Germany).

### 3.2. Plant Material

The aerial parts of *S. tenuipes* were collected during the flowering stage (in June 2015) in Texanna, Algeria (36°40′41.06″ north latitude 5°46′32.73″ longitude east). Note that because *S. tenuipes* is a near threatened species, only a restricted amount of sample was collected, and the procedure was performed under the supervision of the National Park of Taza of Jijel/Algeria. The identification of the sample was done by the botanist M. Sebti from the Laboratory of Biotechnology Environment and Health, University of Jijel and a voucher specimen (ST133) was deposited in the herbarium National Park of Taza. The aerial parts of the plant were dried at room temperature in the absence of direct sunlight and then powdered by grinding.

### 3.3. Extraction

The powdered plant material (500 g) was macerated for 24 h with 80% methanol solution (500 mL) and the extraction procedure was repeated two more times with solvent replacement. The hydro-alcoholic mixture from the extractions was combined and filtered, followed by concentration under reduced pressure to dryness. The obtained residue was dissolved in hot water (500 mL). After filtration, the solution was partitioned with ethyl acetate (1:1) three times, and with *n*-butanol (1:1), three times. The resulting ethyl acetate and *n*-butanol extracted solutions were evaporated to dryness, giving rise to the respective dried fractions (EA and Bu, respectively).

### 3.4. Characterization of Phenolic Compounds

#### 3.4.1. Total Phenolic Content

Total phenolic compounds were quantified by the method described by Müller et al. [48] with slight modification. Briefly, 20 µL of the fraction (or gallic acid), 100 µL of Folin–Ciocalteu reagent (diluted 1:10 ratio with distilled water), and 75 µL of sodium carbonate were added to a 96-well microplate. The absorbance was measured at 765 nm after 2 h in a microplate reader (PerkinElmer, Inc, Waltham, MA, USA). The total phenolic content was expressed as mg of gallic acid equivalents (GAE) per g of fraction.

#### 3.4.2. Total Flavonoid Content

Estimation of the total flavonoid concentration was based on the aluminum nitrate method described by Park et al. [49] with some modifications. Briefly, 50 μL of plant fraction (or quercetin), 130 μL MeOH, 10 μL of 1 M potassium acetate, and 10 μL of 10% aluminum nitrate were mixed in each well of a microplate. Upon 40 min, the absorbance was read in a microplate reader (EnSpire Multimode Plate Reader Perkin, Waltham, MA 02451, USA). The total flavonoid content was expressed as mg of quercetin equivalents (QEs) per g of fraction.

#### 3.4.3. UHPLC-ESI-DAD-MS^n^ Analysis

This analysis was performed in an Ultimate 3000 (Dionex Co., San Jose, CA, USA) apparatus equipped with an ultimate 3000 Diode Array Detector (Dionex Co., San Jose, CA, USA) and coupled to a mass spectrometer, following the general procedure previously described [50]. Samples (5 mg/mL) were prepared in ethanol and analyzed through the injection of 2 μL. The chromatographic apparatus consisted of a quaternary pump, an autosampler, a photodiode-array detector, and an automatic thermostatic column compartment. The column used was a 100 mm length, 2.1 mm i.d., 1.9 μm particle diameter, end-capped Hypersil Gold C18 column (Thermo Scientific, San Jose, CA, USA), and its temperature was adjusted to 30 °C. The mobile phase was composed of (A) 0.1% (*v*/*v*) formic acid and (B) acetonitrile. The solvent gradient started with 5% of solvent B, reaching 40% at 14 min and 100% at 16 min, followed by a return to the initial conditions. The flow rate used was 0.2 mL.min^−1^ and UV–Vis spectral data for all peaks were accumulated in the range 200–600 nm. The mass spectrometer used was a Thermo LTQ XL (Thermo Scientific, USA) ion trap MS equipped with an ESI source. Control and data acquisition were carried out with the Thermo X-Calibur Qual Browser data system (Thermo Scientific, USA). Nitrogen above 99% purity was used and the gas pressure was 520 kPa (75 psi). The instrument was operated in the negative-ion mode with the ESI needle voltage set at 5.00 kV and an ESI capillary temperature of 275 °C. The full scan covered the mass range from *m*/*z* 100 to 2000. CID-MS/MS and MS^n^ experiments were simultaneously acquired for precursor ions using helium as the collision gas with a collision energy of 25–35 arbitrary units.

The identification of coumaric acid was performed by comparison of the retention times, absorption spectra, and MS data with the standard. The remaining phenolic compounds were assigned based on their retention times, absorption spectra, and interpretation of the fragmentation pattern, as well as comparison with literature data. Compounds appearing above the limit of quantification were quantified by peak integration, using the external standard method, with the corresponding standard (coumaric acid) or the most structurally related standard compounds available. Kaempferol glucosides were quantified as kaempferol at 340 nm (calibration curve y = 17655x − 35885, R^2^ = 0.998); coumaroyl derivatives were quantified as coumaric acid 320 nm (calibration curve y = 48154x − 20234, R^2^ = 1.000); and harpagoside and acetyl martynoside were quantified as verbascoside at 280 nm (calibration curve y = 11806x − 595, R^2^ = 0.999).

### 3.5. Assessment of Anti-Inflammatory Activity

The anti-inflammatory effect of EA and Bu fractions was assessed in two animal models, namely the carrageenan-induced rat paw edema method and the xylene-induced ear edema test, in addition to the in vitro evaluation of the inhibition of albumin denaturation technique. The animal experiments were approved by the Laboratory Animal Ethics committee of University of Jijel/Algeria/Biology Department (permit number: BDLAEC 2018407). Animals were allowed to acclimatize to the laboratory environment for 7 days prior to the experiment. They were kept in cages at 22 ± 1 °C with free access to pellet food and water and on a 12-h light/dark cycle. Due to restrictions in plant sampling (and inherent mass limitations of EA and Bu fractions), in vivo anti-inflammatory tests could not be preceded by an acute toxicity study. Instead, only one concentration of extract was tested. On the basis of previous works that showed anti-inflammatory-related events for *Scrophularia striata* extracts (tested at 100 and 200 mg/Kg, po, with better results for the latter [51]), we opted to test the fractions at concentrations of 200 mg/Kg, po. Besides, as preliminary studies with EA and Bu fractions at 1 mg/mL showed no activity against albumin denaturation, this test was performed with fractions at 2 mg/mL.

#### 3.5.1. Carrageenan-Induced Rat Paw Edema

Four groups of 6 rats (female Wistar rats of 180–200 g each) were treated by oral administration with EA (200 mg/Kg, po), Bu (200 mg/Kg, po) (EA and Bu were dissolved in saline buffer with 10% DMSO), the drug diclofenac (Dic, 10 mg/Kg, po), or vehicle for the control group (isotonic NaCl solution 0.9%, po). One hour after, edema was induced in the rats by injecting carrageenan 0.1 mL 1% into the subplantar tissue of the right hind paw. Paw volume was measured using a plethysmometer before administering the inflammatory agent and 1, 2, and 3 h after induction of inflammation. Increase of paw volume was measured as the difference in paw volume at the beginning of the experiment, before inducing edema (0 h) and paw volume at the respective hours. The percent inhibition of inflammation was calculated according to the following formula [52]:

Edema inhibition (%) = 100(V_0_ − V_t_/V_0_),

where V_t_ is the volume of the paw of rats given test fraction or the standard drug at the corresponding time and V_0_ is the volume of the paw of rats of the control group at the same time.

#### 3.5.2. Xylene-Induced Ear Edema

The xylene-induced ear edema test was performed as previously described by Tang et al. [53]. Animals were divided into four groups of six rats each. Edema was induced by applying 0.03 mL of xylene to the inner surface of the right ear 30 min after the oral administration of saline (10 mL/Kg, po), EA and Bu fractions (200 mg/Kg, po [51]) (EA and Bu were dissolved in saline buffer with 10% DMSO), or diclofenac (10 mg/Kg, po). The left ear was considered as the control. Two hours after the application of xylene, the rats were sacrificed using chloroform anesthesia and both ears were removed. Circular sections (6 mm diameter) of both the right (treated) and left (untreated) ears were sampled with a punch and weighted [32]. The edematous response was measured as the weight difference between the right and left ears, where the inhibition level was calculated according to the following equation:

Edema inhibition (%) = 100 (V_c_ − V_t_/V_c_),

where V_c_ represents the difference in the weight of the ear in the control and V_t_ the difference in the weight of the ear in the group treated with standard/fractions.

#### 3.5.3. Albumin Denaturation

The anti-inflammatory activity was also evaluated by using the inhibition of albumin denaturation technique, which was carried out as previously described by Sakat et al. [54] with slight modifications. A solution of 0.2% *w/v* of bovine serum albumin (BSA) was prepared in a Tris Buffer Saline and pH was adjusted to 6.6 using HCl. The fractions (2 mg/mL) or diclorofenac (1 mg/mL) were prepared by using DMSO (10%) as a solvent. Afterwards, 0.5 mL of fraction were added to 0.5 mL of BSA solution and heated at 72 °C for 5 min. After cooling, the turbidity of the samples was measured at 660 nm and the percent of inhibition of protein denaturation was calculated as follows:

Denaturation inhibition (%) = A_Control_ − A_Fraction_/A_Control_ × 100,

where A is the absorbance.

### 3.6. Determination of Antioxidant Activity

#### 3.6.1. 2,2 diphenyl-1-picryhydrazyl Free Radical Scavenging Assay

The scavenging activity of the fractions was determined spectrophotometrically by the 2,2 diphenyl-1-picryhydrazyl radical (DPPH^●^) assay described by Blois [55] with slight modification. Firstly, 6 mg of DPPH was dissolved in a volume of 100 mL of methanol and kept away from light. After that, 160 μL of the prepared DPPH methanolic solution and 40 μL of the sample solutions (3.125 µg/mL–200 µg/mL), dissolved in methanol, at different concentrations were mixed in a 96-well microplate. The plate was then incubated in the dark at room temperature for 30 min and the absorbance of the reaction mixture was measured at 517 nm by a 96-well microplate reader. The scavenging capability of DPPH^●^ was calculated using the following equation and the results were given as IC_50_ value (µg/mL), which is the concentration of 50% inhibition:

DPPH radical scavenging (%) = A_control_ − A_sample_/A_control_ × 100,

where A_control_ is the absorbance of the DPPH and A_sample_ is the absorbance of DPPH^●^ in the presence of the sample. BHA and BHT were used as antioxidant standards.

#### 3.6.2. 2,2-azinobis-(3-ethylbenzothiazoline-6-sulfonic acid) (ABTS) Cation Radical Decolorization Assay

ABTS radical scavenging activity was determined according to the method described by Re et al. [56] with slight modifications. The pre-formed radical cation (ABTS^•+^) was produced by the reaction between 7 mM ABTS in H_2_O and 2.45 mM potassium persulfate, kept away from light at room temperature for 12 h. Before usage, the absorbance of the solution obtained was adjusted by methanol to 0.700 ± 0.020 at 734 nm. After that, 160 μL of ABTS^•+^ solution was mixed with 40 μL of plant fraction at different concentrations (3.125 µg/mL–200 µg/mL) and the absorbance was measured at 734 nm after 10 min by a 96-well microplate reader. The ABTS radical scavenging activity was calculated using the following equation and the results were given as IC_50_ value (µg/mL), which is the concentration of 50% inhibition:

ABTS radical scavenging (%) =A_control_ − A_sample_/A_control_ × 100,

where A_control_ is the absorbance of the ABTS^•+^ and A_sample_ is the absorbance of ABTS^•+^ in the presence of the sample. BHA and BHT were used as antioxidant standards.

#### 3.6.3. Cupric -Reducing Antioxidant Capacity (CUPRAC)

Cupric-reducing antioxidant capacity was determined according to the method described by Apak et al. [57] with slight modifications. First, 50 μL of 10 mM cupric chloride, 50 μL of 7.5 mM neocuprine, and 60 μL of ammonium acetate buffer (1 mol/L, pH 7.0) solutions were added to each well (in a 96 well plate) containing 50 μL of fraction at 7 different concentrations in the range of 3.125 to 200 µg/mL. These mixtures were incubated for 1 h at room temperature and measured against blank at 450 nm using a 96-well microplate reader. Results were given as the absorbance (A_0.50_ µg/mL) compared to those of BHA and BHT used as antioxidant standards.

#### 3.6.4. Superoxide Radical (O_2_^•−^) Scavenging Activity

Superoxide radical was accessed by the alkaline DMSO method, using alkaline DMSO as the generating system [58]. Briefly, 130 µL of alkaline DMSO (20 mg NaOH dissolved in 100 mL of DMSO), 30 µL of NBT (1 mg/mL), and 40 µL of fraction at different concentrations between 3.125 and 200 µg/mL were added in a 96-well microplate. The absorbance was measured at 560 nm after 2 min in a 96-well microplate reader. The antioxidant activity was calculated using the following equation and the results were given as IC_50_ value (µg/mL):

% Superoxide scavenging (%) = A _control_ − A _sample_/A_control_ × 100,

where A _control_ is the absorbance of ‘alkaline DMSO^+^NBT’ and A _sample_ is the absorbance of ‘alkaline DMSO ^+^ NBT’ in the presence of the sample. Tannic acid and α-tocopherol were used as antioxidant standards.

### 3.7. Enzyme Inhibitory Activity

#### 3.7.1. α-Amylase

α-Amylase inhibitory activity was performed using the iodine/potassium iodide (IKI) method [59] with slight modifications. First, 25 µL of sample solution at different concentrations in the range between 6.25 and 400 µg/mL was mixed with α-amylase solution (50 µL) in phosphate buffer (pH 6.9 with 6 mM sodium chloride) and then incubated for 10 min at 37 °C. After that, a starch solution (50 µL, 0.1%) was added and incubated for 10 min. Finally, 25 µL of HCl (1M) and 100 µL IKI were added to each well in the 96-well microplate. Similarly, a blank was prepared by adding to the sample solution all reaction reagents except the enzyme (alpha-amylase) solution. The absorbance of both the sample and blank were read at 630 nm while the blank absorbance was subtracted from that of the sample. The pharmacological inhibitor, acarbose, was used as a positive control and the α-amylase inhibitory activity was calculated as follows:

Activity inhibition (%) = A _control_ − A _sample_/A _control_ × 100,

where A is the absorbance.

#### 3.7.2. α-Glucosidase

α-glucosidase inhibitory activity was performed according to the methodology described by Sinéad Lordan et al. [60]. First, 50 µL of sample solution (concentrations between 15.62 µg/mL and 1000 µg/mL) was mixed with 50 µL of 5 mM *p*-nitrophenyl-α-d-glucopyranoside solution (in phosphate buffer) in a 96-well microplate. After 5 min of incubation at 37 °C, phosphate buffer (100 µL) containing 0.1 U/mL α-glucosidase was added to each well. Blank readings (without enzyme) were subtracted from each well and the results were compared to the control. The absorbance of the sample and blank were recorded at 405 nm and acarbose was used as the positive control. The activity of α-glucosidase was evaluated as follows:

Activity inhibition (%) = A _control_ − A _sample_/A _control_ × 100,

where A is the absorbance.

### 3.8. Statistical Analysis

One-way analysis of variance (ANOVA) followed by Tukey’s test were used to detect any significant differences among different means. A *p*-value under 0.05 was assumed to indicate a significant difference. The results were analyzed using GraphPad Prism 6 (GraphPad Software, San Diego, CA, USA).

## 4. Conclusions

The aerial parts of *S. tenuipes* were subjected to successive solvent fractionation and two fractions were targeted, ethyl acetate (EA) and *n*-butanol (Bu), which were clearly distinct regarding their phenolic composition, with EA being particularly rich in the phenylethanoid acetyl martynoside while Bu was mostly represented by the iridoid harpagoside. These differences could be associated to their distinct biological properties: the EA fraction had a marked anti-inflammatory effect, both in vitro and in vivo, while the Bu fraction had superior antioxidant activity. Overall, these findings allow a better understanding of the potential biological properties of the aerial parts of *S. tenuipes* while simultaneously hypothesizing that the phenolic compounds, namely martynoside and harpagoside, may be major contributors to the anti-inflammatory and antioxidant effects of the plant, respectively. Further investigation is running in our lab in order to isolate the mentioned compounds and further elucidate this hypothesis.

## Figures and Tables

**Table 1:** Phenolic compounds of *S. tenuipes* EA and Bu fractions.

Peak	Rt (min)	λ_max_	[M − H]^−^	ESI MS/MS Product Ions	Probable Compound	EA (mg/g)	Bu (mg/g)
1	8.9	294sh, 320	179	135	Caffeic acid	d	-
2	9.9	312	309	187,118,163	Coumaroyl-*O*-rhamnoside	d	d
3	10.5	277, 320	367	349,307,203,161,289,245,191,173	Feruloylquinic acid	d	-
4	10.7	272, 334	609	447, 285,489,429,255	Kaempferol-*O*-dihexoside	-	68.0 ± 4.0
5	11.0	309	163	119	Coumaric acid	21.4 ± 2.5	7.5 ± 0.9
6	12.2	282, 334	447	285,284,327,255; MS^3^ [285]: 267, 241, 185	Kampferol-*O*-hexoside	2.2 ± 0.3	39.3 ± 3.0
7	12.3	313	509	307, 265, 163, 235	Coumaric ac derivative	d	d
8	13.0	272, 331	447	285, 327,255; MS^3^[285]: 267, 241/239	Kaempferol-*O*-hexoside	d	d
9	13.6	313	351	333,229,187,273,163,119	Coumaroyl-*O*-acetyl-rhamnoside (isom 1)	d	d
10	13.8	313	351	187, 229, 333, 163, 119	Coumaroyl-*O*-acetyl-rhamnoside (isom 2)	d	d
11	14.9	314	679	637, 499, 351,619, 229, 333, 273	Coumaroyl-*O*-acetyl-rhamnoside derivative (isom 1)	1.5 ± 0.1	6.8 ± 0.7
12	15.0	314	679	499,351,637,619,333,229,273,517	Coumaroyl-*O*-acetyl-rhamnoside derivative (isom 2)	1.5 ±0.2	7.7 ± 0.9
13	15.3	313	351	163, 187, 333, 119	Coumaroyl-*O*-acetyl-rhamnoside (isom 3)	3.7 ± 0.4	-
14	15.7	281	493	345, 179, 181	Harpagoside (isom 1)	7.2 ± 0.6	40.4 ± 4.0
15	16.4	280	493	345, 179, 201,147	Harpagoside (isom 2)	142.0 ± 15.1	316.0 ± 8.0
16	16.9	277	693	651, 633, 505, 517, 475, 457	Acetyl martynoside	416.5 ± 17.7	90.7 ± 10
					TPC (mg GAE/g)	225.5 ± 0.9	181.4 ± 0.5
					TF (mg QE/g)	21.7 ± 3.2	64.6 ± 4.8

EA, ethyl acetate fraction; Bu, *n-*butanol fraction; isom, isomer; TPCs, Total phenolic compounds estimated by the Folin–Ciocalteu method and expressed as mg of gallic acid equivalents (GAE) per g of fraction; TFs, total flavonoids estimated by the aluminum nitrate method and expressed as mg of quercetin equivalents (QEs) per g of fraction; +, detected; - absent.

**Table 2:** Anti-inflammatory activities of *S. tenuipes* fractions.

Treatment	Paw-Edema Volume (mL)			Ear-Edema Weight (mg)	BSA Denaturation (% Inhibition)
	1 h	2 h	3 h		
Control	2.40 ± 0.01^a^	1.68 ± 0.02^a^	1.40 ± 0.02^a^	4.00 ± 0.03^a^	-
EA	1.19 ± 0.32^b^	0.91 ± 0.09^b^	0.52 ± 0.14^b^	1.90 ± 0.05^b^	80.72 ± 0.75^a^
Bu	1.96 ± 0.21^c^	1.33 ± 0.49^c^	1.08 ± 0.54^c^	3.00 ± 0.07^c^	NA
Diclofenac	0.54 ± 0.05^d^	0.3 ± 0.01^d^	0.25 ± 0.05^d^	1.00 ± 0.05^d^	94.04 ± 0.49^b^

BSA, bovine serum albumin; Bu, *n*-butanol fraction; EA, ethyl acetate fraction; NA—not active. In vivo studies were performed with 200 mg/kg (fractions) or 10 mg/kg (diclofenac); Anti-denaturation effect of BSA was evaluated with 2 mg/mL (fractions) or 1 mg/mL (diclofenac). Values are means ± SEM. Means followed by different letters (^a,b,c,d^) in the same column are significantly different at *p* < 0.05 according to Tukey’s test or T-test (BSA denaturation).

**Table 3:** Antioxidant activities of *S. tenuipes* fractions.

	ABTS^•+^ (IC_50_, µg/mL)	DPPH^•^ (IC_50_, µg/mL)	O^2•–^ (IC_50_, µg/mL)	Cupric Reduction (A_0.50_, µg/mL)
EA	20.3 ± 0.3^a^	111.2 ± 1.4^a^	18.9 ± 1.2^a^	53.4 ± 0.6^a^
Bu	18.7 ± 0.5^b^	69.0 ± 0.2^b^	18.5 ± 2.8^a^	43.3 ± 1.3^b^
Diclofenac	-	-	-	-
BHA	1.8 ± 0.1^c^	5.7 ± 0.4^c^	-	3.6 ± 0.2^c^
BHT	1.3 ± 0.3^d^	22.3 ± 1.2^d^	-	9.6 ± 0.9^d^
α-tocopherol	-	-	˂3.1^b^	-
Tanic acid	-	-	˂3.1^b^	-

EA, ethyl acetate fraction; Bu, *n*-butanol. Values are means of three independent assays ± SD. Means followed by different letters (^a,b,c,d^) in the same column are significantly different at *p* < 0.05 according to Tukey’s test.

**Table 4:** Inhibitory activities of *S. tenuipes* fractions towards α-amylase and α-glucosidase.

	α-Amylase (IC_50_, µg/mL)	α-Glucosidase (IC_50_, µg/mL)
EA	8.3 ± 0.2^a^	≥1000^a^
Bu	11.0 ± 0.5^b^	≥1000^a^
Acarbose	45.2 ± 1.3^c^	275.4 ± 1.6^b^

EA, ethyl acetate fraction; Bu, *n*-butanol fraction; Values are means of three independent assays ± SD. Means followed by different letters (^a,b,c^) in the same column are significantly different at *p* < 0.05 according to Tukey’s test.
